# Epidemiology of Humanpapilloma virus infection among women in Fujian, China

**DOI:** 10.1186/s12889-017-4651-7

**Published:** 2017-08-03

**Authors:** Conglian Wu, Xianjin Zhu, Yanli Kang, Yinping Cao, Pingxia Lu, Wenjuan Zhou, Hong Zhou, Yang Zhang, Yanfang Song

**Affiliations:** 1 Department of Laboratory Medicine, Fujian Medical University Union Hospital, 29 Xinquan Road, Fuzhou, 350001 China; 2 Department of Laboratory Medicine, Affiliated Renmin Hospital of Fujian University of Traditional Chinese Medicine; Fujian province key Laboratory of Integrated Traditional and Western Immunity Nephrology, 602 Bayiqi Road, Fuzhou, 350001 China; 3 Department of Laboratory Medicine, The First Hospital of Quanzhou Affiliated to Fujian Medical University, 248-252 East Street, Quanzhou, Fujian 362000 China

**Keywords:** Human papillomavirus (HPV), Prevalence, Genotyping, Cervical cancer, Fujian

## Abstract

**Background:**

Human papillomavirus (HPV) infection is the main etiological factor for the development of cervical cancer. Here we assessed the prevalence and distribution of HPV genotypes in Fujian population.

**Methods:**

A total of 8678 women aging from 17 to 84 years olds were recruited from the Fujian Medical University Union Hospital in Fujian Province. Every woman had a face-to-face interview. Cervical samples were collected from each participant and HPV screening was conducted using microarray hybridization.

**Results:**

Our study showed that the HPV prevalence in Fujian province was 38.3%. Among the positive individuals, 70.6% were detected for single HPV infection, 29.4% for multiple HPV infections. Further analysis showed that the prevalence of HPV infection significantly increased from 2009 to 2015. The four most common high risk human papillomavirus (HR-HPV) genotypes were HPV16 (8.5%), HPV52 (7.9%), HPV58 (6.2%), HPV 53 (3.5%), collectively accounting for 60.5% of all detected HPV infection. Age subgroup analysis showed two peaks for the frequencies of overall and multiple HPV infections, one for the group of women under 25 years old, and the other for the group over 55 years old.

**Conclusions:**

HPV infection is becoming serious in Fujian province, which indicates the imperative to implement a HPV vaccination and screening program for this region.

## Background

Cervical cancer is the fourth most common cancer among women worldwide [1]. In China, cervical cancer is the second most common gynecologic cancers among women, with an increasing incidence and mortality rates in young women [2]. The relationship between cervical cancer and human papillomavirus (HPV) infection is widely studied, and it has been approved that HPV infection plays an important role in the development of cervical cancer during past decades [3, 4], so it is necessary for the cervical cancer prevention to detect timely, prevent and decrease HPV infection. So far more than 100 HPV genotypes have been identified and about 40 genotypes can infect the genital tract [5]. According to their associations with cervical cancer and precancerous lesions, HPV genotypes are generally classified into high-risk (HR) and low-risk (LR) genotypes [5]. More than 15 HR-HPV genotypes are associated with the development of cervical cancer, of which HPV 16, 18, 31, 33, 35, 45, 52, and 58 are associated with 90% of invasive cervical cancers and HPV 16 and 18 are associated with 70% of invasive cervical cancer [6, 7], The LR-HPV genotypes including HPV 6, 11, 42, 43 and 44, are associated with hyperplasic lesions [5]. Considering that the important role of HPV infection in the development of cervical cancer and other associated diseases, HPV screening is strongly advised and has an important prognostic or therapeutic value because it is more sensitive and cost-effective than cytology-based screening for detection of cervical cancer [8–12]. HPV screening, especial HR-HPV screening, is useful in the selection of those patients who are at increased risk for cervical cancer and may therefore provide timely prevention and treatment [8, 13].

Nowadays, vaccine is crucial in the prevention of HPV infection and cervical cancer. Recently, three licensed HPV vaccines have been implementation in most western countries. Cervarix® vaccine is a bivalent HPV vaccine against HPV16 and 18, Gardasil® vaccine is a quadrivalent vaccine against HPV 6, 11, 16, and 18, and Gardasil 9 vaccine would target four types (HPV 6, 11, 16, and 18), and five new HPV types (HPV 31, 33, 45, 52, and 58) [14–16]. Although, three licensed HPV vaccines are shown to have the potential to significantly reduce the burden of cervical cancer, there remain the possibility that non-vaccine HPV genotypes may replace HPV vaccine genotypes as causal agents of cervical precancer and cancer in vaccinated populations [6]. Previous studies have found that HPV prevalence and genotype distribution vary greatly with geographic areas [17]. Thus, it is necessary to survey the prevalence and distribution of HPV genotypes in Fujian province to provide the baseline information on HPV infection status before the available of vaccines.

To date, in Fujian province, there is only one previous report about the prevalence and distribution of HPV infection among 2338 women in 2008–2009 [18], and the available data are limited and outdated. For obtaining a more current dataset, we conducted a study to investigate the overall, age-specific and genotype-specific prevalence of HPV infection among women in this region. Our study would provide guidance for the implement of HPV vaccination programs.

## Methods

### Study population

The study population was originally obtained from the outpatient services of the departments of gynecology, health medical examination of the Fujian Medical University Union Hospital in Fujian Province from January 2009 to December 2015. Every woman attending the gynecological examination had a face-to-face interview. Inclusion criteria were designed for enrolled women who: a) had a history of current or past sexual activity, b) had an intact uterus, c) had no use of vaginal medication or washing presently, d) were physically and mentally competent, e) were not presently pregnant, f) were a permanent resident of the local area, g) were willing to undergo an HPV test and participate in the present study. Finally, 76 samples were excluded because they were duplicate samples from the same women (in this study the first sample was used).Thus, the final study population included 8678 eligible women (age range: 17–84 years; mean age: 39 years). These studies were performed in accordance with ethical guidelines under the protocols approved by the Institutional Medical Ethics Review Board of Fujian Medical University Union Hospital, Fuzhou, China.

### Sample collection and HPV genotyping

Cervical samples were obtained from women using a cytobrush and used for genomic DNA extraction. Subsequently, HPV DNA was detected and genotyped by flow-through hybridization and gene chip by HybriMax (Chaozhou Hybribio Limited Corporation, Chaozhou, China), in accordance with the manufacturer’s instructions. This kit can capture the special HPV DNA strain via oligonucleotide probes immobilized in a nylon membrane. This technology can detect 21 HPV genotypes, including 6 low-risk (LR) genotypes (6, 11, 42, 43 and 44, CP8304), and 15 high-risk (HR) genotypes (16, 18, 31, 33, 35, 39, 45, 51, 52, 53, 56, 58, 59, 66, 68). Simultaneously, the positive and negative controls were used to validate the HPV test.

### Statistical analysis

Statistical analysis was performed using SPSS software for windows (version 18.0). We calculate the prevalence of HPV infection by dividing the HPV positive number by total number of samples that were successfully tested for HPV. The binomial 95% confidence interval (95% CI) for HPV prevalence was calculated. In addition, we used linear-by-linear association test to analyze the linear relationship between the HPV prevalence and time trends. *P*-value <0.05 was considered as statistical significance.

## Results

### The overall prevalence of HPV infection

A total of 8678 women were involved in this study, of which 3328 (38.3%) were positive for HPV infection. Single HPV infection accounted for 27.1% (2351/8678) of all the participants and 70.6% (2351/3328) of positive samples, and further analysis showed that, among women with single HPV infection, single HR-HPV infection accounted for 59.5% (1982/3328), while single LR-HPV accounted for 11.1% (369/3328). Multiple HPV infections accounted for 11.3% (977/8678) of all the participants and 29.4% (977/3328) of positive samples, and further analysis showed that, among women with multiple HPV infections, 21.2% (704/3328) were detected for double infections, 5.4% (179/3328) for triple infections, and 2.8% (94/3328) for four or more infections.

### Time trends in prevalence of HPV infection

We analyzed the prevalence of HPV infection in Fujian province from 2009 to 2015 and found that the prevalence of HPV infection significantly increased, ranging from 29.4% (95% CI 25.8–32.6%) in 2009 to 43.4% (95% CI 40.9–46.1%) in 2015 (Table 1). Further analysis showed that, from 2009 to 2015, the prevalence of HPV 39, 51, 52, 53, 56, 59, and 68 significantly increased over time, while the prevalence of other HPV genotypes did not change significantly (Table 2).

**Table 1:** Prevalence of HPV in women from Fujian, China

Year	Age Median (rang)	HPV positive (n)	HPV negative (n)	Total (n)	Prevalence (%)	95% CI of infection rate (%)
2009	37 (18–68)	209	503	712	29.4	25.8–32.6
2010	37 (17–71)	268	516	784	34.2	31.0–37.4
2011	38 (15–76)	403	614	1017	39.6	36.7–42.7
2012	39 (18–84)	499	799	1298	38.4	35.7–41.1
2013	40 (15–82)	578	905	1483	39.0	36.4–41.7
2014	41 (13–80)	758	1215	1973	38.4	36.3–40.3
2015	41 (12–77)	613	798	1411	43.4	40.9–46.1
Total	39 (12–84)	3328	5350	8678	38.3	37.3–39.4

**Table 2:** The HPV genotype distribution in Fujian province from 2009 to 2015

HPV type	Year							Total	Frequency for all sample (%)	Frequency for positive sample (%)	95% CI for all samples (%)
	2009 N(%)	2010 N(%)	2011 N(%)	2012 N(%)	2013 N(%)	2014 N(%)	2015 N(%)				
16	57(8.0)	70(8.9)	87(8.6)	124(9.6)	119(8.0)	164(8.3)	121(8.6)	742	8.5	22.3	8.0–9.1
18	14(2.0)	22(2.8)	28(2.8)	30(2.3)	34(2.3)	51(2.6)	37(2.6)	216	2.5	6.5	2.2–2.8
31	12(1.7)	18(2.3)	26(2.6)	24(1.8)	34(2.3)	39(2.0)	20(1.4)	173	2.0	5.2	1.7–2.3
33	16(2.2)	22(2.8)	40(3.9)	32(2.5)	42(2.8)	4(1.7)	37(2.6)	223	2.6	6.7	2.2–2.9
35	3(0.4)	5(0.6)	10(1.0)	6(0.5)	13(0.9)	11(0.6)	18(1.3)	66	0.8	2.0	0.6–0.9
**39**	**5(0.7)**	**10(1.3)**	**18(1.8)**	**23(1.8)**	**33(2.2)**	**66(3.3)**	**24(1.7)**	**179**	**2.1**	**5.4**	**1.8–2.4**
45	2(0.3)	2(0.3)	4(0.4)	8(0.6)	10(0.7)	5(0.3)	10(0.7)	41	0.5	1.2	0.3–0.6
**51**	**9(1.3)**	**4(0.5)**	**4(0.4)**	**3(0.2)**	**23(1.6)**	**47(2.4)**	**58(4.1)**	**148**	**1.7**	**4.4**	**1.4–2.0**
**52**	**36(5.1)**	**47(6.0)**	**84(8.3)**	**84(6.5)**	**123(8.3)**	**172(8.7)**	**143(10.1)**	**689**	**7.9**	**20.7**	**7.4–8.5**
**53**	**15(2.1)**	**14(1.8)**	**34(3.3)**	**42(3.2)**	**58(3.9)**	**71(3.6)**	**72(5.1)**	**306**	**3.5**	**9.2**	**3.1–3.9**
**56**	**4(0.6)**	**13(1.7)**	**6(0.6)**	**18(1.4)**	**18(1.2)**	**28(1.4)**	**39(2,8)**	**126**	**1.5**	**3.8**	**1.2–1.7**
58	33(4.6)	38(4.8)	67(6.6)	81(6.2)	97(6.5)	135(6.8)	84(6.0)	535	6.2	16.1	5.7–6.7
**59**	**5(0.7)**	**8(1.0)**	**8(0.8)**	**15(1.2)**	**13(0.9)**	**19(1.0)**	**30(2.1)**	**98**	**1.1**	**2.9**	**0.9–1.3**
66	10(1.4)	13(1.7)	17(1.7)	26(2.0)	31(2.1)	31(1.6)	42(3.0)	170	2.0	5.1	1.7–2.2
**68**	**8(1.1)**	**8(1.0)**	**14(1.4)**	**35(2.7)**	**27(1.8)**	**43(2.2)**	**37(2.6)**	**172**	**2.0**	**5.2**	**1.7–2.3**
6	22(3.1)	17(2.2)	17(1.7)	28(2.2)	37(2.5)	39(2.0)	37(2.6)	197	2.3	5.9	2.0–2.6
11	18(2.5)	28(3.6)	27(2.7)	27(2.1)	43(2.9)	33(1.7)	18(1.3)	194	2.2	5.8	1.9–2.5
42	3(0.4)	0(0.0)	4(0.4)	7(0.5)	9(0.6)	6(0.3)	37(2.6)	66	0.8	2.0	0.6–1.0
43	0(0.0)	0(0.0)	0(0.0)	0(0.0)	0(0.0)	3(0.2)	33(2.3)	36	0.4	1.1	0.3–0.6
44	4(0.6)	2(0.3)	8(0.8)	6(0.5)	7(0.5)	11(0.6)	0(0.0)	38	0.4	1.1	0.3–0.6
CP8304	7(1.0)	20(2.6)	36(3.5)	53(4.1)	74(5.0)	63(3.2)	57(4.0)	310	3.6	9.3	3.2–4.0

Bold indicates a statistically significant

### Genotype-specific prevalence of HPV infection

**Fig. 1:**
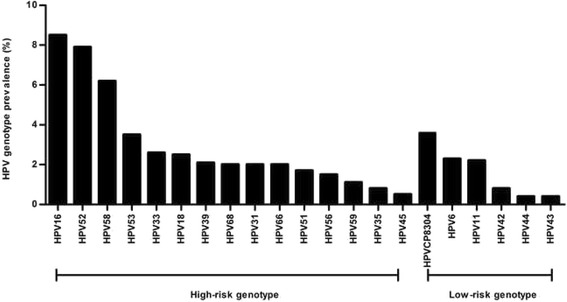
Distribution of different HPV genotypes in Fujian province. HPV genotypes, including 6 low-risk (LR) genotypes (6, 11, 42, 43 and 44, CP8304), and 15 high-risk (HR) genotypes (16, 18, 31, 33, 35, 39, 45, 51, 52, 53, 56, 58, 59, 66, 68) were detected by flow-through hybridization and gene chip by HybriMax

A total of 21 HPV genotypes were identified among Fujian women in this study. The prevalence of HR-HPV infection were 33.9% (2940/8678), substantially higher than that of LR-HPV infection (8.9%, 773/8678). The most prevalent HR-HPV genotype was HPV 16 (8.5%), followed by HPV 52 (7.9%), HPV 58 (6.2%), and HPV 53 (3.5%). In addition, the most common LR-HPV genotypes were HPV 8304 (3.6%), HPV 6 (2.3%) and HPV 11 (2.2%) (Fig. 1).

### Age-specific prevalence of HPV infection

**Fig. 2:**
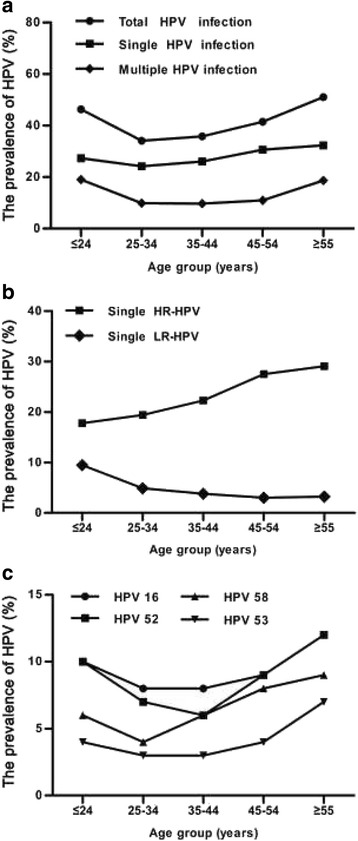
Specific age-related prevalence of HPV infection. **a** The age-specific prevalence of total HPV, single HPV, and multiple HPV. **b** The age-specific prevalence of single HR-HPV, and single LR-HPV. **c** The age-specific prevalence of the four most prevalent HR-HPV genotypes (HPV 16, 52, 58 and 53) in Fujian province

In this study, all the participants were stratified into 5 groups based on their age. (Table 3). Age-specific prevalence of total HPV infection exhibited a “U-shaped” curve, with the frequencies of 46.3% (95% CI 42.3–50.4%), 34.1% (95% CI 32.1–36.0%), 35.8% (95% CI 34.1–37.4%), 41.5% (95% CI 39.3–43.8%), and 51.0% (95% CI 47.2–54.9%) in different age groups, respectively. The first peak was appeared in the group under ≤24 years of age (46.3%; 263/568). Values decreased to 34.1% in the group 25–34 years of age and contiguously increased to 51.0% in the group ≥55 years of age (≥55; 51.0%) (Fig. 2a). The lowest prevalence (19%, 792/2322) was found in the 25–34 age groups. Similar age-prevalence curve was also observed in multiple HPV infections and single HPV infection (Fig. 2a). However, the prevalence of single HR-HPV exerted an increasing trend with age, and single LR-HPV infection was highest in less than 24 years age group and then decreased significantly with advancing age (Fig. 2b). We analyzed the age-specific prevalence of the prevalent HR-HPV genotypes (HPV 16, 52, 58 and 53) in Fujian province, and found that the bimodal age distribution was also observed in HPV 16 and 52 but not for HPV 58 and 53 (Fig. 2c).

**Table 3:** Specific age-related HPV genotype distribution

HPV type	Age group (year)					HPV positive (N)	Frequency for all sample (%)	Frequency for positive sample (%)	95% CI for all samples (%)
	≤ 24 N(%)	25 ~ 34 N(%)	35 ~ 44 N(%)	45 ~ 54 N(%)	≥ 55 N(%)				
16	56(9.9)	180(7.8)	259(7.9)	172(9.0)	75(12.2)	742	8.6	22.3	8.0–9.1
18	17(3.0)	46(2.0)	79(2.4)	54(2.8)	20(3.2)	216	2.5	6.5	2.2–2.8
31	14(2.5)	36(1.6)	70(2.1)	36(1.9)	17(2.8)	173	2.0	5.2	1.7–2.3
33	12(2.1)	46(2.0)	78(2.4)	59(3.1)	28(4.5)	223	2.6	6.7	2.2–2.9
35	4(0.7)	16(0.7)	26(0.8)	11(0.6)	9(1.5)	66	0.8	2.0	0.6–0.9
39	13(2.3)	47(2.0)	62(1.9)	39(2.0)	18(2.9)	179	2.1	5.4	1.8–2.4
45	3(0.5)	6(0.3)	12(0.4)	12(0.6)	8(1.3)	41	0.5	1.2	0.3–0.6
51	14(2.5)	31(1.3)	49(1.5)	38(2.0)	16(2.6)	148	1.7	4.4	1.4–2.0
52	54(9.5)	173(7.5)	209(6.4)	180(9.4)	73(11.9)	689	7.9	20.7	7.4–8.5
53	24(4.2)	65(2.8)	106(3.2)	68(3.6)	43(7.0)	306	3.5	9.2	3.1–3.9
56	7(1.2)	33(1.4)	29(0.9)	35(1.8)	22(3.6)	126	1.5	3.8	1.2–1.7
58	32(5.6)	101(4.3)	197(6.0)	147(7.7)	58(9.4)	535	6.2	16.1	5.7–6.7
59	25(4.4)	11(0.5)	27(0.8)	19(1.0)	16(2.6)	98	1.1	2.9	0.9–1.3
66	17(3.0)	45(1.9)	62(1.9)	27(1.4)	19(3.1)	170	2.0	5.1	1.7–2.2
68	9(1.6)	42(1.8)	63(1.9)	41(2.2)	17(2.8)	172	2.0	5.2	1.7–2.3
6	53(9.3)	57(2.5)	54(1.7)	21(1.1)	12(1.9)	197	2.3	5.9	2.0–2.6
11	52(9.2)	62(2.7)	47(1.4)	22(1.2)	11(1.8)	194	2.2	5.8	1.9–2.5
42	7(1.2)	16(0.7)	16(0.5)	16(0.8)	11(1.8)	66	0.8	2.0	0.6–1.0
43	5(0.9)	10(0.4)	9(0.3)	7(0.4)	5(0.8)	36	0.4	1.1	0.3–0.6
44	5(0.9)	8(0.3)	11(0.3)	6(0.3)	8(1.3)	38	0.4	1.1	0.3–0.6
CP8304	33(5.8)	68(2.9)	111(3.4)	64(3.4)	34(5.5)	310	3.6	9.3	3.2–4.0

## Discussion

It has been demonstrated that HPV, particularly HR-HPV types, is major causative factors in the development of cervical cancer [3, 19]. However, HPV genotypes may exhibit differing distributions according to geographic region. In this study, we investigated the prevalence of HPV infection in Fujian province, China.

Our results showed that the overall prevalence of HPV infection in Fujian province was 38.3%. HPV infection rate in Fujian province was significantly higher than that in Henan (22.8%), Zhejiang (13.3%), and a previous study in Fujian province (22.5%), but was similar to that in Harbin (36.5%) [18, 20–22]. Considering the same laboratory methods method (flow-through hybridization methods) used for HPV infection detection in these reports, the difference in the prevalence of HPV infection might primarily be attributed to differences in the geographical regions. In additional, survey period may be part of the reason: survey period in our study is 2009–2015, and survey period in Luoyang, Zhejiang, Harbin is 2012–2013 [22], 2006–2008 [20], May to December of 2011 [21], respectively. Importantly, the prevalence of HPV infection in Fujian province increased significantly, ranging from 29.4% in 2009 to 43.4% in 2015. These results suggested that HPV infection in Fujian province has reached the level of “HPV-heavy-burden” and the infection rate increased with years. The possible reason for increase HPV prevalence over time in Fujian province was that, with the economic development and the increase of international communication since the late 1990’s in Fujian province, the view of people toward sexual behavior was really beginning to change. The number of sexual partners has increased in young women and the age of sexual debut become earlier over time. Such increased sexual activities may increase the exposure of HPV, thus potentially lead to an increase over the years in prevalence of HPV. In addition, the absence of the prevention and regular screening of HPV infection also lead to an increased prevalence of HPV infection. However, the exact reasons for the increase of HPV prevalence in Fujian province still remain unclear. Considering the heavy burden of HPV infection, it is imperative to implement HPV screening and vaccines program for this region.

The analysis for genotype-specific prevalence of HPV infection shown that the most common HR-HPV genotype was HPV 16, followed by HPV 52, 58, 53, and 33, whereas HPV 18 was in a relatively low prevalence. The most common LR-HPV genotype was HPV CP8304, followed by 6, 11, 42 and 43. The results in this study were different from most previous studies in other region of china. For example, in Harbin province, main genotypes are HPV 16, 52, 58, 18 and 45 [21]. In Luoyang province, main genotypes are HPV 16, 58, 33, 56 and 35 [22]. And in Zhejiang province, main genotypes are HPV 52, 16, 58, 68, 81 [20]. These studies show that HPV 16 plays a predominant role in all regions, but other prevalent genotypes vary in different regions. Accordingly, the research for genotypes-specific prevalence of HPV infection in various regions would provide guidance for developing more effective HPV vaccine for these regions. In addition, our results were also different from previous report of Fujian, which showed that the top 5 HPV genotypes were HPV 52, 16, 18, 33 and 53 [18]. We speculate that this difference may be caused by study period.

Several studies have shown that HPV 52 and HPV 58 are more prevalent genotypes and predominantly associated with the development of cervical cancer in Asian [23–25]. In China, particularly in southern and coastal regions, these two genotypes are found to be more prevalent among women with cervical cancer and precancerous lesion compared to other regions [26]. In this study, we found that the prevalence of HPV 52 and HPV 58 was ranked second and third in Fujian province. Interestingly, in this study, we found that the prevalence of HPV 53 infection in Fujian province was ranked fourth. This result was not very common in other researches and revealed the special distribution of HR-HPV in Fujian population. It has been reported that HPV 53 is associated with cervical carcinoma [18]. Considering that the vaccines are types-specific, current HPV vaccines cannot provide complete protection against non-vaccine HPV genotypes. When considering a vaccine, the data from this study show that Cervarix vaccine (targets HPV 16, 18) and Gardasil vaccine (targets HPV 6, 11, 16, 18) only cover 27.9% and 37.8% of infections, respectively. When considering Gardasil 9 vaccine (targets HPV 6, 11, 16, 18, 31, 33, 45, 52, 58), the potential for HPV prevention would rise to 74.2% in Fujian women.

Previous studies in China have observed that HPV prevalence has two peaks in terms of age, while the peak age varied among different studies [8, 27, 28]. The first peak is generally found in younger women, usually in their early twenties, and the other peak usually appear in menopausal women. In agreement with previous studies, our study found that the age-specific prevalence of total HPV, single HPV and multiple HPV all formed an approximate U-shaped curve with two peaks, one at the ≤24 age group and the other at the ≥55 age group. The first peak observed in younger women (≤24 years old) may account for sexual activity [29] and immature immunity to HPV [30], thus, it is the optimum time to offer HPV vaccines to adolescent females before sexual debut and HPV exposure. HPV infection rate was decreased to the lowest at the 25–34 age groups. This phenomenon might attribute mostly to their mature immunity to HPV, which have greater ability to prevent and clear HPV infections. In addition, women in the 25–34 age groups were generally during the reproductive age, a period in which some of women are more likely to visit the gynecologic clinic for reasons linked to reproduction, therefore they have higher probability to be “healthy”. The second peak in older women may account for HPV persistence and reactivation of latent viruses due to physiologic and immunologic dysregulation during menopausal transition [31]. Considering that the older women with HPV infection are prone to have viral persistence [32, 33], they may have higher risk for the development of cervical cancer. Accordingly, comprehensive, efficient and regular cervical screening programs should be implemented for older women with HPV infection.

Some studies have reported that multiple infections are frequently detected in different grades of cervical cancer [34]. In this study, we found that multiple HPV infections accounted for 29.4% of all infections. Our finding is similar to the previous studies which multiple infection accounts for 26–38% of all infections in China [35, 36]. The role of multiple HPV infections on cervical cancer remains debatable. Some studies have reported that multiple infections are associated with an increased risk of precancerous lesions and cervical cancer [37, 38]. However, other studies reported that the risk of developing cervical cancer in women with multiple HPV infections is no greater than for those with single HPV infection [39, 40]. Therefore, the potential role of multiple infections in cervical cancer needs further investigation.

There were several limitations to this study. The study population was originally obtained from the outpatient services of the departments of gynecology and health medical examination of the Fujian Medical University Union Hospital. To overcome this shortcoming, all women who visited cervical cancer screening and the Health Medical Examination Center from January 2009 to December 2015 were enrolled in study population, but for consecutive females who attend the center, we only collected the HPV results of the first examination, and the next HPV test results were excluded. In the future, a population-based study should be performed to investigate the HPV infection. In addition, the cytological data on the enrolled individuals were not collected in this study, thus, we did not have the data of cervical lesions classification, which may impact on base prevalence.

## Conclusions

Our study provides the data on the prevalence and distribution of HPV infection in Fujian province, the southeast of China. Our results suggest that HPV infection in Fujian province has reached the level of “HPV-heavy-burden” and increased with years. The four most frequent HR-HPV genotypes is HPV 16, 52, 58, 53. Our results would provide baseline information for estimating HPV vaccines and screening for this region.

## Abbreviations

HPV: Human papillomavirus
HR: High-risk
HR-HPV: High risk human papillomavirus
LR: Low risk
LR-HPV: Low risk human papillomavirus
